# Intensity Dependent Effects of Interval Resistance Training on Myokines and Cardiovascular Risk Factors in Males With Obesity

**DOI:** 10.3389/fendo.2022.895512

**Published:** 2022-06-10

**Authors:** Ali Ataeinosrat, Ayoub Saeidi, Hossein Abednatanzi, Hiwa Rahmani, Asieh Abbassi Daloii, Zhaleh Pashaei, Vida Hojati, Gholam Basati, Ali Mossayebi, Ismail Laher, Michaela G. Alesi, Anthony C. Hackney, Trisha A. VanDusseldorp, Hassane Zouhal

**Affiliations:** ^1^ Department of Physical Education and Sport Science, Science and Research Branch, Islamic Azad University, Tehran, Iran; ^2^ Department of Physical Education and Sport Sciences, Faculty of Humanities and Social Sciences, University of Kurdistan, Sanandaj, Iran; ^3^ Department of Sport Sciences and Health, Shahid Beheshti University, Tehran, Iran; ^4^ Department of Exercise Physiology, Ayatollah Amoli Branch, Islamic Azad University, Amol, Iran; ^5^ Department of Exercise Physiology, Faculty of Physical Education and Sport Sciences, University of Tabriz, Tabriz, Iran; ^6^ Department of Biology, Damghan Branch, Islamic Azad University, Damghan, Iran; ^7^ Department of Clinical Biochemistry, Faculty of Medicine, Ilam University of Medical Sciences, Ilam, Iran; ^8^ Department of Kinesiology, College of Health Sciences, University of Texas at El Paso, El Paso, TX, United States; ^9^ Department of Anesthesiology, Pharmacology, and Therapeutics, The University of British Columbia, Vancouver BC, Canada; ^10^ Department of Exercise Science and Sport Management, Kennesaw State University, Kennesaw, GA, United States; ^11^ Department of Exercise & Sport Science; Department of Nutrition, University of North Carolina, Chapel Hill, NC, United States; ^12^ Univ Rennes, M2S (Laboratoire Mouvement, Sport, Santé), Rennes, France; ^13^ Institut International des Sciences du Sport (2I2S), Irodouer, France

**Keywords:** cardiovascular risk factor, myokines, interval resistance training, exercise, obesity

## Abstract

**Objective:**

To determine the effects of different intensities of interval resistance training (IRT) protocols on the levels of select myokines (decorin, follistatin, myostatin, activin A, transforming growth factor beta-1 [TGF-β1]), and cardiometabolic and anthropometric measures in males with obesity.

**Methods:**

Forty-four obese males (age: 27.5 ± 9.4 yr.; height: 165.4 ± 2.8 cm; weight: 97.9 ± 2.6 kg and BMI: 35.7 ± 4.3 kg/m^2^) were randomly assigned to one of four groups (n=11 per group): low-intensity interval resistance training (LIIRT), moderate-intensity interval resistance training (MIIRT), high-intensity interval resistance training (HIIRT) or control (C). The LIIRT group performed 10 exercises in 3 sets of 40% (20 repetitions), the MIIRT group performed 10 exercises in three sets of 60% (13 repetitions), and the HIIRT group performed 10 exercises in three sets of 80% (10 repetitions) of one maximum repetition (1RM), which were followed with active rest of 20% of 1RM and 15 repetitions. The resistance training groups exercised ~70 min per session, 3 days per week, for 12 weeks. Measurements were taken at baseline and after 12 weeks of exercise training.

**Results:**

Baseline levels of myokines, cardiovascular risk factors, anthropometry, body composition, and cardio-respiratory fitness were not different between the four groups (p>0.05). The group x time interactions for decorin, activin A, follistatin, myostatin, and TGF-β1, total cholesterol (TC), triglyceride (TG), high-density cholesterol (HDL), low-density cholesterol (LDL), anthropometry, body composition, and cardio-respiratory fitness were statistically significant (p<0.05). There were increases in post-test values for decorin, follistatin, HDL (p<0.05) and decreases in TC, TG, TGF-β1, LDL, and myostatin levels in the LIIRT, MIIRT, and HIIRT groups compared to pretest values (p<0.05). Changes in fat mass, VO_2peak,_ HDL, TG, glucose, activin A, decorin were not significant in LIIRT compared to the control group, while changes in activin A, follistatin, and TFG-β1 levels were greater in HIIRT and MIIRT groups compared to the LIIRT group (p<0.05).

**Conclusion:**

The LIIRT, MIIRT, and HIIRT protocols all produced beneficial changes in decorin, activin A, follistatin, myostatin, and TGF-β1 levels, and cardiometabolic risk factors, with greater effects from the MIIRT and HIIRT protocols compared to LIIRT.

## Introduction

Obesity is associated with atherosclerosis, hypertension, type 2 diabetes, gastrointestinal disorders (1) and also decreased muscle mass, and the development of sarcopenia, which increases the risk of diabetes (2). Circulating insulin levels are increased in many patients with type 2 diabetes to compensate for muscle cell dysfunction and to maintain glucose levels (3). Several body tissues play critical roles in regulating metabolism, health, and muscle growth pathways by secreting hormones that affect the comorbidities associated with obesity; for example, adipose tissue secretes adipokines, the liver secretes hepatokines, and muscles secrete myokines (4).

Myokines, such as IL-6, myostatin, irisin, myonectin, and decorin, are peptides produced and released by myocytes that affect the physiology of muscles and other organs (4). For example, myostatin, an important myokine in the development and regulation of hypertrophy, is a transforming growth factor belonging to the TGF-β family (5). Myostatin is also produced and secreted from adipose tissue and heart muscle (6, 7). The myostatin gene, type IIB activator receptor, and some of its inhibitors, such as follistatin (FST) and follistatin -3-like (FSTL3), are expressed in adipose tissue (8). Interestingly, the number of fat cells is reduced in mice whose myostatin gene expression was inhibited in adipose tissue (6). Although the reasons for the association between myostatin gene expression and lipid cell depletion are not clear, this action can reduce or prevent the differentiation and proliferation of preadipocyte cells (6). FST is a potent myostatin antagonist and a member of the TGF-β family of proteins and glycoproteins produced by skeletal muscle cells. FST prevents muscle wasting by preventing myostatin from binding to its receptor (9, 10). Another member of the TGF-β family is TGF-β1, which increases adipose tissue synthesis and myostatin levels by stimulating Smad3 signaling (11). Levels of TGF-β1 correlate with body mass index (BMI), fat mass, and oxygen uptake (VO_2_) in obese people. TGF-β1 levels are higher in overweight (BMI of 25- 29.9 kg/m^2^) and obese (BMI> 30 kg/m^2^) than in healthy individuals (BMI less than 24.9 kg/m^2^). There is also an inverse correlation between TGF-β1 levels and maximal VO_2_ (12), as well as a positive correlation between TGF-β1 levels and adiposity in rodents and humans. Further, systemic blockade of TGF-β signaling protects mice from obesity, diabetes, and hepatic steatosis. Together, these results demonstrate that TGF-β signaling regulates glucose tolerance and energy homeostasis (12). The myokine decorin is part of the extracellular matrix secreted during skeletal muscle contraction (2, 13) and plays a vital role in cell growth by modulating growth factor activity (14, 15). Decorin expression in adipose tissue is increased in obese patients and has been proposed to mediate adipose tissue activity in obesity (16). More specifically, decorin can act as a receptor for resistin on adipocyte precursors and affect adipocyte metabolism and proliferation (17). Furthermore, the expression of decorin mRNA in adipose tissue in obese and overweight individuals suggests a role for decorin in angiogenesis or vascular homeostasis in adipose tissue (18, 19).

Various regular exercise training protocols are recommended as non-pharmacological therapies to reduce obesity-related disorders (20). The secretion of adipomyokines such as TGF-β1, myostatin, FST, IL-7, and decorin from the extracellular matrix of adipose or muscle tissue is regulated by exercise (12, 21). Interval training (IT) is characterized by brief, repeated work intervals interspersed with periods of recovery (e.g., low-intensity exercise). Depending on the training intensity, intense bouts can last from a few seconds to several minutes, with activities separated by a few minutes of rest with low-intensity activity (22). When continuous endurance training is compared to IT using the same workload or energy cost, IT can be an alternative to continuous endurance training because of similar or even superior effects on health-related markers in healthy and unwell individuals (23–25). In addition, IT produces similar physiological adaptations as moderate-intensity continuous exercise (22), which can be important from a public health perspective (26–28). Furthermore, recent evidence suggests that IT is more enjoyable for some participants than continuous moderate-intensity exercise (29). On the other hand, resistance training is recommended by the American Association of Sports Medicine as an integral part of the exercise training protocols (30). Resistance activity, which involves activities that increase the mechanical load on muscles, is specifically designed to increase muscle strength and endurance (31), basal metabolic rate (32), and decrease body fat mass, lipid profile, and insulin sensitivity (33).

Considering the health effects of resistance training and increasing interest in performing IT (22), it is important to study the health effects of interval resistance training (IRT). IRT emphasizes multi-joint muscle groups and improves the lipid profile and some adipokines more than traditional and circuit resistance training (34), although it can be argued that the protocol of IRT could differentially regulate decorin, FST, TGF-β1, and myostatin levels. To our knowledge, there are no reports on the effects of IRT on the levels of these muscle growth indices. Intensity is the most important component of resistance training and can have a multilateral effect. Intense exercise training can stimulate muscle injury and inflammatory factors, and has been reported to be more effective than low-intensity physical activity on improving metabolic status. However, one study reported no differences between the intensities of 85, 75, and 50% of 1RM on lipid and insulin profiles (35), while another study reported that low-intensity resistance training did not affect myostatic and myogenic factors, but that intense resistance training improved muscle development by increasing follistatin and decreasing myostatin levels (36).

We compared the effects of three intensities of exercise (low (40%), medium (60%), and high (80%) of 1 RM) to examine the hypothesis that changes in myokine levels and cardiovascular risk factors is related to the intensity of resistance interval training (IRT) in men with obesity. We determined the effects of IRT on the levels of select myokines (decorin, follistatin, myostatin, activin A, TGF-β) and cardiometabolic and anthropometric measures in males with obesity.

## Methods

### Participants

Forty-four obese males (age: 27.5 ± 9.4 yr; height: 165.4 ± 2.8 cm; weight: 97.9 ± 2.6 kg) were recruited for this study through advertisements using posters, emails, and social media applications. The criteria for inclusion were: ages 23 - 32 years, BMI >30 kg·m^−2^, waist-to-height ratio (WHR) > 0.6, sedentary (less than 1 hour of physical activity per week during the last year), an absence of diagnosed illnesses (e.g., cardiovascular disease, diabetes, and hypertension), and if they were non-smokers, not receiving hormonal or mental therapy, and were not consuming alcohol. Participants consuming dietary supplements and drugs that affected the muscle or adipose tissue metabolism (e.g., amino acids, beta-blockers, beta-agonists, calcium channel blockers, and corticosteroids) were excluded from the study. A physician evaluated these criteria using the Physical Activity Readiness Questionnaire (PAR-Q) and medical health/history questionnaires (37).

All participants read and signed an informed consent form. The study procedures were reviewed and approved by the Research and Ethics Committee of the Islamic Azad University (Ethics code: IR-IAU1397-16). All procedures were performed according to the latest revision of the Declaration of Helsinki.

### Study Design

**Figure 1:**
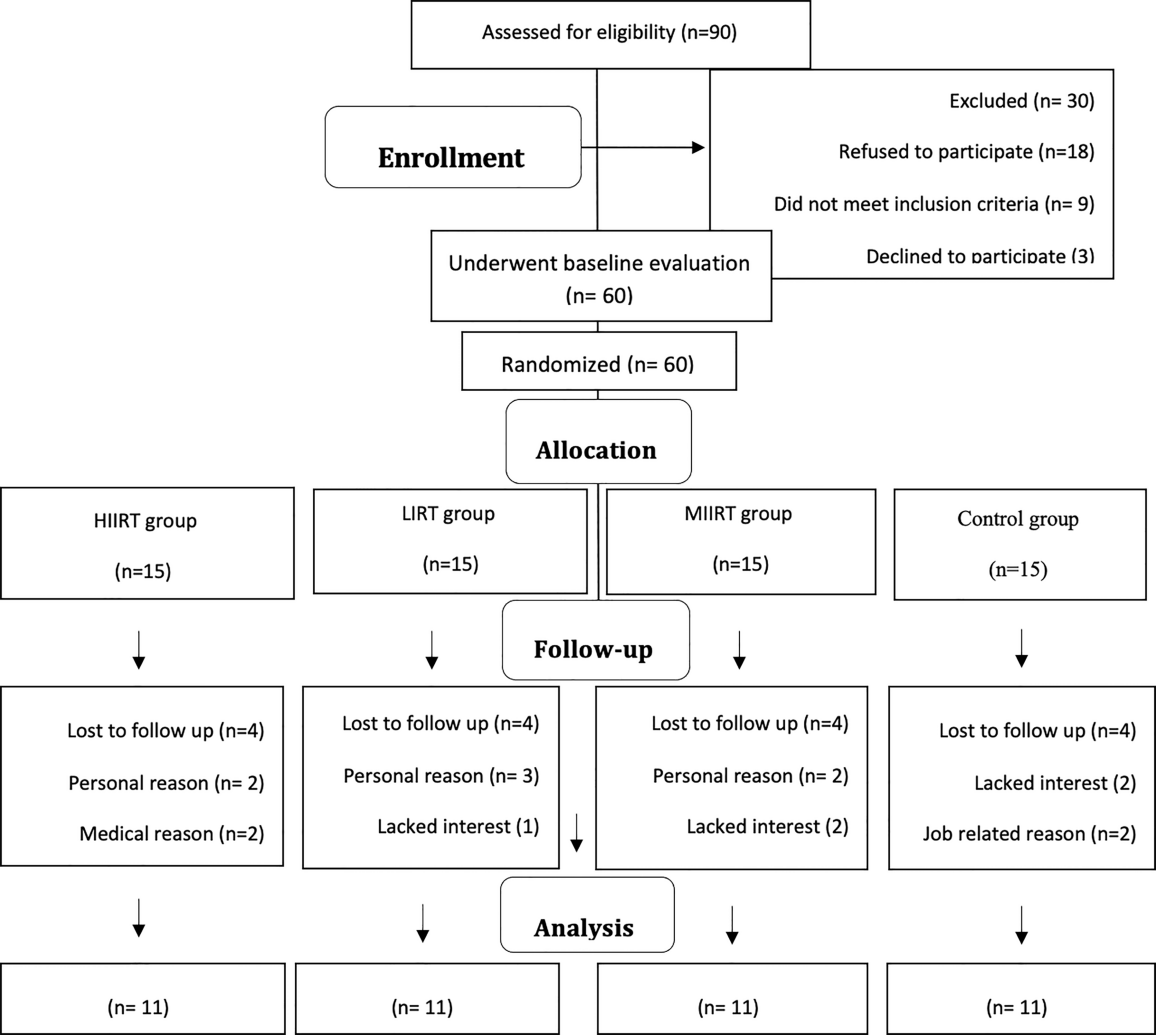
Study flow chart.

Before obtaining baseline measurements, each participant was thoroughly acquainted with all testing and procedures. Participants were subsequently randomly assigned into one of four groups (all n=11): low-intensity interval resistance training (LIIRT), moderate-intensity interval resistance training (MIIRT), and high-intensity interval resistance training (HIIRT), or control (C). Participants in the control group were instructed to maintain their usual lifestyles (inactive) for 12 weeks. Study measurements were collected at two time points: a) baseline [48 hours before starting the preparatory phase of the study] and b) after 12 weeks [48 hours after the last training session]. Study measurements were made at the same time of day (within ~1 hour) and under the same environmental conditions (~20°C and ~55% humidity). The participants were asked not to modify their regular lifestyles and dietary habits for the duration of the study period **(****Figure 1****)**.

### Anthropometry, Body Composition, and Cardio-Respiratory Fitness

Anthropometric and body composition measures (body weight, height, BMI) were evaluated using standard techniques before and after the training protocol (38). Barefoot standing height (cm) was measured using a wall-mounted stadiometer (SECA^®^, UK), while body weight was measured on a digital scale (SECA^®^, UK) with the participants wearing light clothing. Height and weight were used to calculate BMI. A bio-impedance analyzer (BIA) (Medigate Company Inc., Dan-dong Gunpo, Korea) was used to assess fat mass and muscle mass. There is acceptable validity (>0.85) and correlation (r=0.73–0.96) of BIA results with reference methods such as underwater weighing, Air displacement plethysmography (ADP), and dual-energy x-ray absorptiometry (DXA) that has been validated in healthy persons (39), athletes (40), patients (41) and obese children (42).

A modified Bruce protocol (43) using a motorized treadmill (H/P/Cosmos, Pulsar med 3p- Sports & Medical, Nussdorf-Traunstein Germany), with an acecptable correlation (r=0.72) with standard Bruce protocol (43, 44), and a gas analyzer system (Metalyzer 3B analyzer, Cortex: biophysik, GMbH, Germany) was used to estimate peak oxygen uptake (VO_2peak_) in a temperature-controlled room (21-23˚ C). The modified Bruce protocol included: five exercise periods in 3-minute stages in which the slope grade progressively increased after the first stage (0, 5, 10, 12, 14%), and the speed started at 2.4 (km/h) and increased after the third stage (2.5, 3.4) (45). Physiological criteria that were used to determine VO_2peak_ following the American College of Sports Medicine (ACSM) guidelines) included: 1) physical exhaustion and a maximal effort (according to Borg scale 6-20; 2) if the supervisor recognized the participants had limiting symptoms based on guidelines for cardiopulmonary exercise testing of the ACSM and American Heart Association (AHA); 3) a plateau in VO_2_ and respiratory exchange ratio (RER) ≥.1.10; and 4) heart rate of more than 90% of a maximal heart rate according to their age-predicted maximum heart rate (46, 47). Blood pressure (Advantage, Model No-6021, American Diagnostic Corporation, Hauppauge, NY, USA) and heart rate (Polar T31TM transmitter, Polar Electro, Kempele, Finland U) were monitored throughout the test.

### Blood Sampling and Analysis

Blood samples (~20 mL) were obtained from the antecubital vein using standard procedures following a 12-hour overnight fast. These blood samples were obtained at baseline and 48 hours after the final training session. Blood samples were centrifuged at 3000 rpm for 10 minutes, and the plasma was stored at -70° C for future analysis. Plasma total cholesterol (TC) and triglyceride (TG) were measured by enzymatic methods (CHOD-PAP); high-density cholesterol (HDL-C) and low-density cholesterol (LDL-C) were determined using a photometric method (Pars Testee’s Quantitative Detection kit, Tehran, Iran) with a coefficient and sensitivity of 1.8% and 1 mg/dl and 1.2% and 1 mg/dl respectively. Insulin levels were measured with an ELISA kit (Demeditec, Germany) with a sensitivity of 1 ng/ml and with between, within-coefficients of variation of 5.1% and 8.4% respectively. Glucose levels were measured with a colorimetric enzymatic kit (Colorimetric Enzymatic Kit, Parsazmun, Tehran, Iran) with a sensitivity of 5 mmol/l. Values of HOMA-IR (IR) were calculated using the formula: homeostasis model analysis (HOMA), HOMA-IR = (22.5 μmol/fasting plasma insulin) X (fasting plasma glucose) (48). Plasma follistatin (R&D Systems, USA. Catalogue No: DFN00. Sensitivity: 83 pg/mL. Intra-CV = 2.7%, inter-CV = 9.2%), Myostatin (R&D Systems, USA. Catalogue No: DGDF80. Sensitivity: 5.32 pg/mL. Intra-CV = 5.4%, inter-CV = 6%), TGF-β (R&D Systems, USA. Catalogue No: DB100B. Sensitivity: 15.4 pg/mL. Intra-CV = 2.9%, inter-CV = 9.1%) concentrations were measured using enzyme-linked immunosorbent assay kits (ELISA). Quantification of plasma decorin was determined *via* human decorin DuoSet ELISA (R&D Systems, USA, Catalogue No. DY143). Plasma activin was measured by ELISA (R&D Systems DAC00B) with intra-assay and inter-assay coefficients of variation of less than 5%.

### Nutrient Intake and Dietary Analysis

Three-day food records (two weekdays and one weekend day) were obtained before and after the study to assess changes in habitual dietary intake over time (49). Each food item was individually entered into Diet Analysis Plus version 10 (Cengage, Boston, MA, USA), and total energy consumption and the amount of energy derived from proteins, fats, and carbohydrates were determined (**Table 1**).

**Table 1:** Mean ( ± SD) values of nutritional intake in the four study groups.

	Control		LIIRT		MIIRT		HIIRT	
	Pre	Post	Pre	Post	Pre	Post	Pre	Post
**Energy (kcal/d)**	2256 ± 67	2262 ± 86	2275 ± 111	2133 ± 150	2253 ± 127	2162 ± 167	2272 ± 177	2111 ± 186
**CHO (g/d)**	281 ± 12.4	283 ± 19.3	277.4 ± 77.1	261 ± 67.5	284 ± 48.6	265 ± 19.2	289 ± 18.6	262 ± 19.1
**Fat (g/d)**	82.2 ± 10.0	81 ± 8.8	86.5 ± 11.7	75 ± 13.2	81.4 ± 14.4	73.1 ± 13.2	80.8 ± 10.87	71.2 ± 15.3
**Protein (g/d)**	104 ± 11.0	106 ± 13.3	101 ± 14.5	95 ± 11.6	103 ± 17.8	93 ± 12.7	104 ± 15.5	90 ± 14.5

LIIRT, low-intensity interval resistance training; MIIRT, moderate-intensity interval resistance training; HIIT, high-intensity interval resistance training.

### Strength Testing

Measurements of body composition, VO_2peak_, and blood sampling were performed on all participants. After performing these tests, all participants were taught back squats, lat pull-down, leg press, chest press, leg extension, leg curls, lateral raise, standing calf raise, biceps curl, and triceps pushdown movements in two sessions. A one-repetition maximum (1-RM) estimation was performed to determine the training intensity for the resistance training (RT) protocol (50, 51) and was repeated at four-week intervals. Before testing, research personnel explained the purpose of each test, risks, possible discomforts, and the participant’s responsibilities. All participants were instructed to refrain from consuming alcohol (for 48 hours) (although non-alcoholic consuming individuals were also included in the study), caffeinated drinks (for 12 hours), and food (for 2 hours) before testing sessions, although ad libitum consumption of water was allowed. Following a light warm-up, with both general and specific components, participants performed strength testing for all exercises included in the RT protocol (back squats, lat pull-down, leg press, chest press, leg extension, leg curls, lateral raise, standing calf raise, biceps curl and triceps pushdown) using variable resistance machines (Hoist Equipment, San Diego, USA). The participants performed two attempts, and their highest lifted weight and number of repetitions were recorded. The number of repetitions to fatigue did not exceed ten. There was a 5 minutes rest period between attempts. Maximal strength was estimated from these assessments using the Brzycki equation: 1RM= weight/(1.0278–0.0278×reps) (52).

### Preparatory Phase

All participants performed one week of RT, consisting of three exercise sessions, to allow for familiarization before the main training intervention. This phase allowed instruction related to correct lifting techniques, familiarization with all exercises and equipment, and ensuring that the participants entered the study at a comparable starting level.

### Resistance Training Protocols

All RT sessions consisted of 70 minutes of exercise that included 10 minutes warm-up, 10 minutes cool down, and about 50 minutes of the main exercise protocol **(** **Table 2** **)**. Exercises in the three resistance training groups were similar and included back squats, lat pull-down, leg press, chest press, leg extension, leg curls, lateral raise, standing calf raises, biceps curl, triceps pushdown.

**Table 2:** Exercises, sets, repetitions, and rest intervals between exercises and sets and a load for each weekly session in the LIIRT, MIIRT, and HIIRT groups.

	LIIRT	MIIRT	HIIRT
**12 Week**			
**Sessions**	**Exercises:** back squats, lat pull-down, leg press, chest press, leg extension, leg curls, lateral raise, standing calf raises, biceps curl, and triceps pushdown **Sets:** 3 **Repetitions:** 20 **Rest interval between exercises:** active rest with 20% of 1RM and 15 repetitions **Load:** 40% of 1RM	**Exercises:** back squats, lat pull-down, leg press, chest press, leg extension, leg curls, lateral raise, standing calf raises, biceps curl, and triceps pushdown **Sets:** 3 **Repetitions:** 13 **Rest interval between exercises:** active rest with 20% of 1RM and 15 repetitions **Load:** 60% of 1RM	**Exercises:** back squats, lat pull-down, leg press, chest press, leg extension, leg curls, lateral raise, standing calf raises, biceps curl, and triceps pushdown **Sets:** 3 **Repetitions:** 10 **Rest interval between exercises:** active rest with 20% of 1RM and 15 repetitions **Intensity:** 80% of 1RM

LIIRT, low-intensity interval resistance training; MIIRT, moderate-intensity interval resistance training; HIIT, high-intensity interval resistance training.

**LIIRT:** Every session for the LIIRT group included 10 exercises with 40% of 1 RM, and 20 repetitions that were followed by active rest of 20% of 1RM and 15 repetitions; participants performed each exercise in 3 sets.

**MIIRT:** Every session for the MIIRT group included 10 exercises with 60% of 1 RM, and 13 repetitions that were followed by active rest of 20% of 1RM and 15 repetitions; participants performed each exercise in 3 sets.

**HIIRT:** Every session for the HIIRT group included 10 exercises with 80% of 1 RM, and 10 repetitions that were followed by active rest of 20% of 1RM and 15 repetitions; participants performed each exercise in 3 sets.

The non-periodized RT protocols were adapted from previous studies (34, 50). During the study, the control group did not participate in any exercise training. Exercises were performed on variable resistance machines (Hoist Equipment, San Diego, USA), and the RT protocol volumes were calculated using the formula:

RT volume  =  the number of sets   ×  repetitions    ×    lifted the weight.

### Statistical Analyses

SPSS (version 22) software was used to analyze all data and the Shapiro–Wilk test was used to evaluate data for normality. An ANOVA repeated measures test was used to compare baseline data of the four groups and to compare changes in the different variables between groups. Differences indicated by ANOVA (p<0.05) and statistical significances were identified using Bonferroni *post-hoc* tests (p<0.05). All data are expressed as mean ± SD. The sample size was designed to detect a difference in study variables, with a 95% confidence interval (CI) and 80% or greater power value. Partial eta-squared (η2) was used to determine effect size.

## Results

### Anthropometry, Body Composition, and Cardio-Respiratory Fitness

There were no differences in body weight (p =0.69), BMI (p =0.52), body fat (p =0.14), free-fat mass (FFM) (p =0.71), and VO_2peak_ (p=0.99) at baseline in the four groups. Group × time interaction was significant for the weight (p=0.0001, η^2^ = 0.37). Pre-post changes in body weight were significant in LIIRT, MIIRT, and HIIRT (p<0.0001) but not in group C (p=0.37). *Post-hoc* analysis revealed that weight changes after training were greater in all training groups compared to the control group (p<0.0001), but the differences between the three intensity groups in weight changes were not significant (p>0.05). Group × time interactions were significant for BMI (p=0.0001, η2 = 0.50). Post-test changes in BMI compared to pretest values in LIIRT, MIIRT, and HIIRT were significant (p<0.05) but not in the control group (p=0.37). *Post-hoc* analysis revealed that the differences between BMI changes after training were significant in all groups compared to the control group (p<0.001), but the differences between different intensities were not significant (p>0.05). Group × time interaction was significant for fat percent (p=0.0001, η^2^ = 0.51). Fat percent reduction in post-test compared to pre-test values was significant in all groups (p<0.05) except in the control group (P=0.98). *Post-hoc* analysis showed that compared to the control group, the significant changes in fat percent occurred after 12 weeks in MIIRT (p<0.001) and HIIRT (p<0.001) groups, but not in the LIIRT group (p=0.26). The difference in fat percent between LIIRT and HIIRT was significant (p=0.001), while the differences between HIIT and MIIRT (p=0.41) and also MIIRT and LIIRT were not significant (p=0.07). The group × time interaction was significant for FFM (p=0.0001, η^2^ = 0.56). Post-test increases of FFM in LIIRT, MIIRT, and HIIRT were significant compared to pre-test values (p<0.05), while the reduction in the control group was not significant (p=0.52). *Post-hoc* analysis showed that the changes of FFM after 12 weeks were significant in all groups compared to the control group (p<0.001), while differences between LIIRT and MIIRT (p=0.18), HIIRT and MIIRT (p=0.99) and also HIIRT and LIIRT (p=0.12) were not significant. The group × time interaction was significant for VO_2peak_ (p =0.0001, η^2^= 0.69). Pre-post changes of VO_2peak_ were not significant for both control (p=0.35) and LIIRT (p=0.53), while the changes were significant for MIIRT (p=0.001) and HIIRT (p=0.001) groups. *Post-hoc* analysis of VO_2peak_ showed that the differences between the control group and MIIRT (P<0.001), control group and LIIRT (p<0.001), LIIRT and MIIRT (p<0.001), and LIIRT and HIIRT were significant (p<0.001), while the differences between MIIRT and HIIRT (p=0.70) and control group and LIIRT (p=0.69) were not significant (**Table 3**).

**Table 3:** Mean ( ± SD) values of glucose, insulin, lipid profile, body composition, and VO_2peak_ for four groups.

	Control		LIIRT		MIIRT		HIIRT	
	Pre	Post	Pre	Post	Pre	Post	Pre	Post
**Height (cm)**	166.5 ± 3.7	–	166.1 ± 2.7	–	164.2 ± 2.6	–	165.2 ± 1.7	–
**Weight (kg)**	97.7 ± 3.8	97.41 ± 2.9	97.9 ± 2.85	95.1 ± 2.9^a,b^	97.4 ± 1.9	94.9 ± 2.4^a,b^	98.7 ± 1.9	95.3 ± 2.4^a b,ab^
**Fat (%)**	32.0 ± 1.0	32.0 ± 1.1	31.1 ± 0.7	30.0 ± 0.8^a,b^	31.4 ± 1.1	28.7 ± 0.8^a,b^	31.9 ± 1.0	28.3 ± 1.0^a,b,ab^
**BMI (kg/m^2^)**	35.3 ± 2.5	35.1 ± 2.2	35.4 ± 1.5	34.4 ± 1.6^a,b^	36.1 ± 1.3	35.2 ± 1.4^a,b^	36.1 ± 0.8	34.9 ± 1.0^a,b,ab^
**FFM (kg)**	26.2 ± 1.0	26.0 ± 1.4	26.5 ± 1.5	28.0 ± 1.1^a,b^	25.9 ± 1.1	28.1 ± 1.3^a,b^	26.4 ± 1.7	28.8 ± 0.9^a,b,ab^
**VO_2peak_** **(mL·kg^-1^·min^-1^)**	26.0 ± 2.4	25.8 ± 2.2	26.2 ± 2.9	26.4 ± 2.7^a^	26.2 ± 2.4	28.7 ± 2.2^a,b^	26.0 ± 1.9	29.0 ± 2.0^a,b,ab^
**HDL (mg/dl)**	39.3 ± 1.2	38.4 ± 1.3	38.8 ± 1.2	39.7 ± 3.9^a^	38.6 ± 1.7	44.5 ± 1.3^a,b^	38.5 ± 1.41	44.8 ± 2.1^a,b,ab^
**LDL (mg/dl)**	125.2 ± 4.4	125.0 ± 4.7	125.5 ± 5.4	121.4 ± 5.2^a,b^	126.7 ± 4.38	111.2 ± 2.9^a,b^	127.1 ± 3.6	110.5 ± 2.5^a,b,ab^
**TC (mg/dl)**	226.7 ± 5.2	226.8 ± 5.2	227.4 ± 5.4	222.0 ± 5.1^a,b^	227.8 ± 5.2	207.4 ± 4.9^a,b^	227.3 ± 5.4	205.2 ± 4.7^a,b,ab^
**TG (mg/dl)**	242.1 ± 4.3	242.2 ± 4.1	245.8 ± 5.9	242.6 ± 4.7^a^	244.5 ± 7.4	215.0 ± 5.3^a,b^	242.9 ± 4.1	212.8 ± 4.6^a,b,ab^
**Insulin (ng/ml)**	18.1 ± 0.6	19.1 ± 0.5	18.8 ± .07	17.6 ± 0.5^a,b^	18.8 ± 0.4	16.1 ± 0.4	19.1 ± 0.4	15.5 ± 0.5^a,b,ab^
**Glucose (mg/dl)**	96.4 ± 13.1	93.7 ± 6.4	98.9 ± 10.7	84.7 ± 4.5^a,b^	99.2 ± 5.7	74.0 ± 5.4	101.6 ± 7.1	71.5 ± 7.7^a,b,ab^
**HOMA-IR**	4.4 ± 0.7	4.2 ± 0.3	4.5 ± 0.4	3.6 ± 0.2^a^	4.6 ± 0.2	2.9 ± 0.2	4.7 ± 0.3	2.7 ± 0.3^a,b,ab^

LIIRT, low-intensity interval resistance training; MIIRT, moderate-intensity interval resistance training; HIIT, high-intensity interval resistance training; BMI, Body Mass Index; FFM, Fat-Free Mass; HDL, High-density lipoprotein; LDL, Low-density lipoprotein; TC, Total cholesterol; TG, Triglyceride; HOMA-IR, Homeostatic Model Assessment of Insulin Resistance.

a Indicates significant differences compared to the Pre-values (p<0.05).

b Significant differences compared to the control group (p<0.05).

^ab^Significant interaction between time and groups (p<0.05).

### Lipid Profiles

There were no differences between the baseline levels of HDL (p=0.57), LDL (p=0.71), TC (p=0.97), and TG (p=0.48) in the four groups. There were significant group × time interactions for HDL (p=0.0001, η^2^ = 0.73), LDL (p=0.0001, η^2^ = 0.86), TC (p=0.0001, η^2^ = 0.96), and TG (p=0.0001, η^2^ = 0.95). Post-test values of HDL increased significantly for both MIIRT (p=0.001) and HIIRT (p=0.001) compared to pre-test values, but the changes in LIIRT (p=0.15) and control group (p=0.13) were not significant. *Post-hoc* analysis of changes in HDL after 12 weeks of resistance training showed that the differences between MIIRT and HIIRT (p=0.96) and LIIRT with the control group (p=0.16) were not significant, while differences between the control group and MIIRT (p<0.001), control group and HIIRT (p<0.001), HIIRT and LIIRT (p<0.001) were significant.

In comparison to thepre-test, post-test values of LDL, TC, and TG decreased in LIIRT, MIIRT, and HIIRT (p<0.0001), while changes in these variables were not significant in the control group (p>0.05). *Post-hoc* analysis of LDL, TC, and TG showed that all differences between LIIRT, MIIRT, and HIIRT with the control group were significant (p<0.001), except for the differences between LIIRT and control groups in TG which were not significant (p=0.10). The differences between MIIRT and HIIRT were not significant for LDL, TC, and TG (p>0.05), while other pairwise differences between LIIRT, MIIRT, and HIIRT were significant for LDL, TC, and TG (p<0.001) (**Table 3**).

### Glucose, Insulin, and HOMA

There were no differences in baseline levels of glucose (p=0.66), insulin (p=0.53), and HOMA (p=0.49) in the four groups. There were significant group X time interactions for insulin (p=0.0001, η^2^ = 0.78), glucose (p=0.0001, η^2^ = 0.49) and HOMA (p=0.0001, η^2^ = 0.69). In comparison to pre-test values, post-test changes in insulin were significant for LIIRT (p=0.001), MIIRT (p=0.001), and HIIRT (p=0.001) groups, but not in the control group (p=0.21). The same pattern of pre-post changes was also observed for glucose (Control: p=0.069, LIIRT: p=0.001, MIIRT: p=0.001, and HIIRT: p=0.001) and HOMA (Control: p=0.17, LIIRT: p=0.001, MIIRT: p=0.001, and HIIRT: p=0.001). *Post-hoc* analysis of differences between groups showed that after 12 weeks of resistance training, insulin (p<0.001) and HOMA (p<0.001) changed significantly in LIIRT, MIIRT, and HIIRT in comparison to the control group. The changes in glucose for control group were different from the changes in MIIRT (p=0.001) and HIIRT (p=0.001) but not with LIIRT (p=0.21). Differences between MIIRT and HIIRT for changes in glucose, insulin, and HOMA were not significant (p>0.05), while there were significant differences between intensity groups (LIIRT, MIIRT, and HIIRT) for changes in glucose, insulin, and HOMA levels (p<0.001) (**Table 3**).

### Decorin, Activin A, Follistatin, Myostatin, and TGF-β1

**Figure 2:**
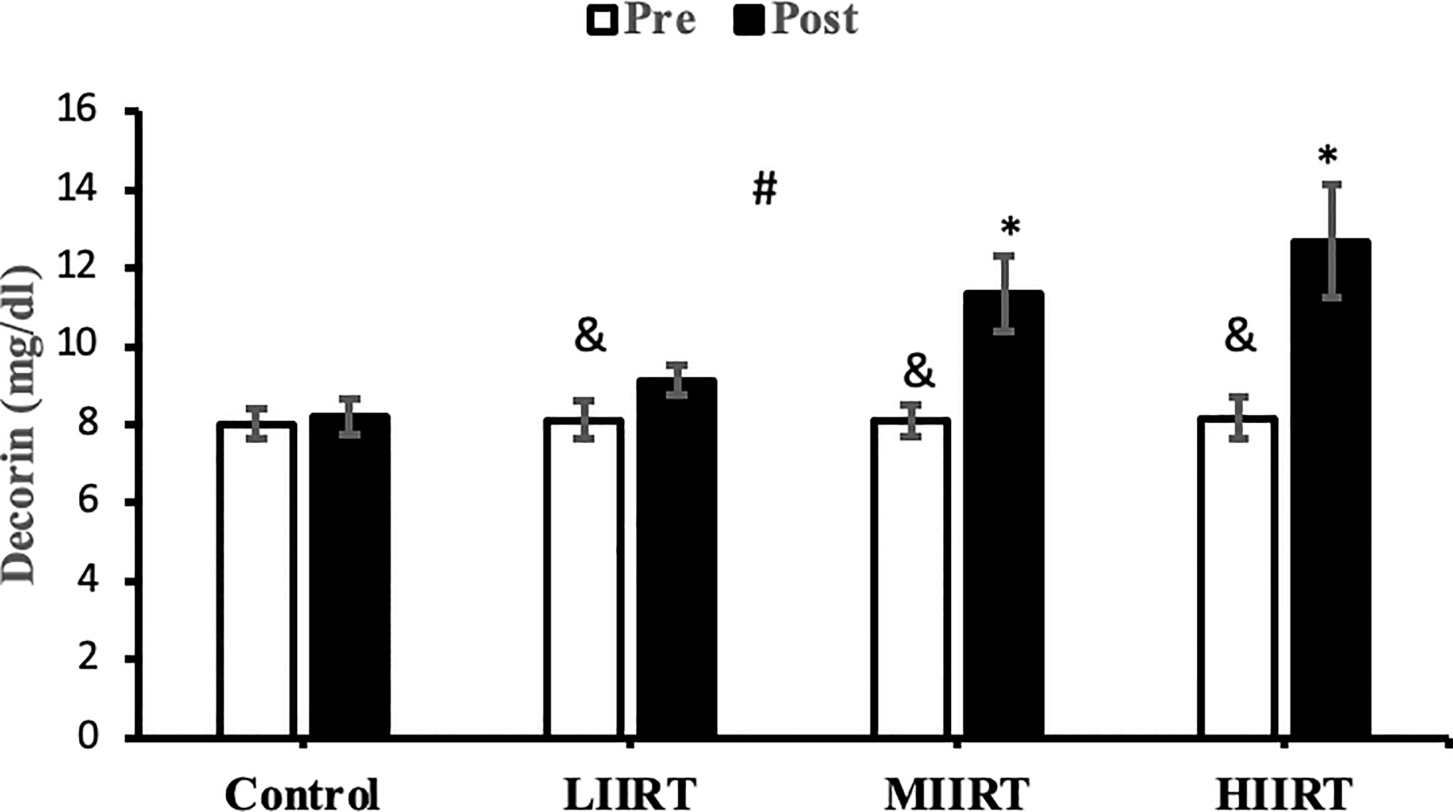
Pre and post-training values (mean ± SD) for decorin in control, low-intensity interval resistance training (LIIRT), moderate-intensity interval resistance training (MIIRT), high-intensity interval resistance training (HIIRT) groups. *Significant differences from the control group (p<0.05). ^&^Significant differences from pretest values in LIIRT, MIIRT, HIIRT (P<0.05). ^#^Significant differences between training groups (p<0.05).

**Figure 3:**
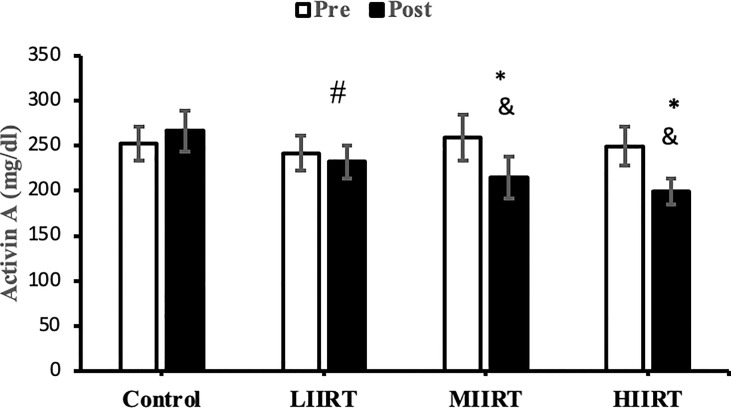
Pre and post-training values (mean ± SD) for activin A in control, low-intensity interval resistance training (LIIRT), moderate-intensity interval resistance training (MIIRT), high-intensity interval resistance training (HIIRT) groups. *Significant differences from the control group (p<0.05). ^&^Significant differences from pretest values (p<0.05). ^#^Significant differences between training groups (p<0.05).

**Figure 4:**
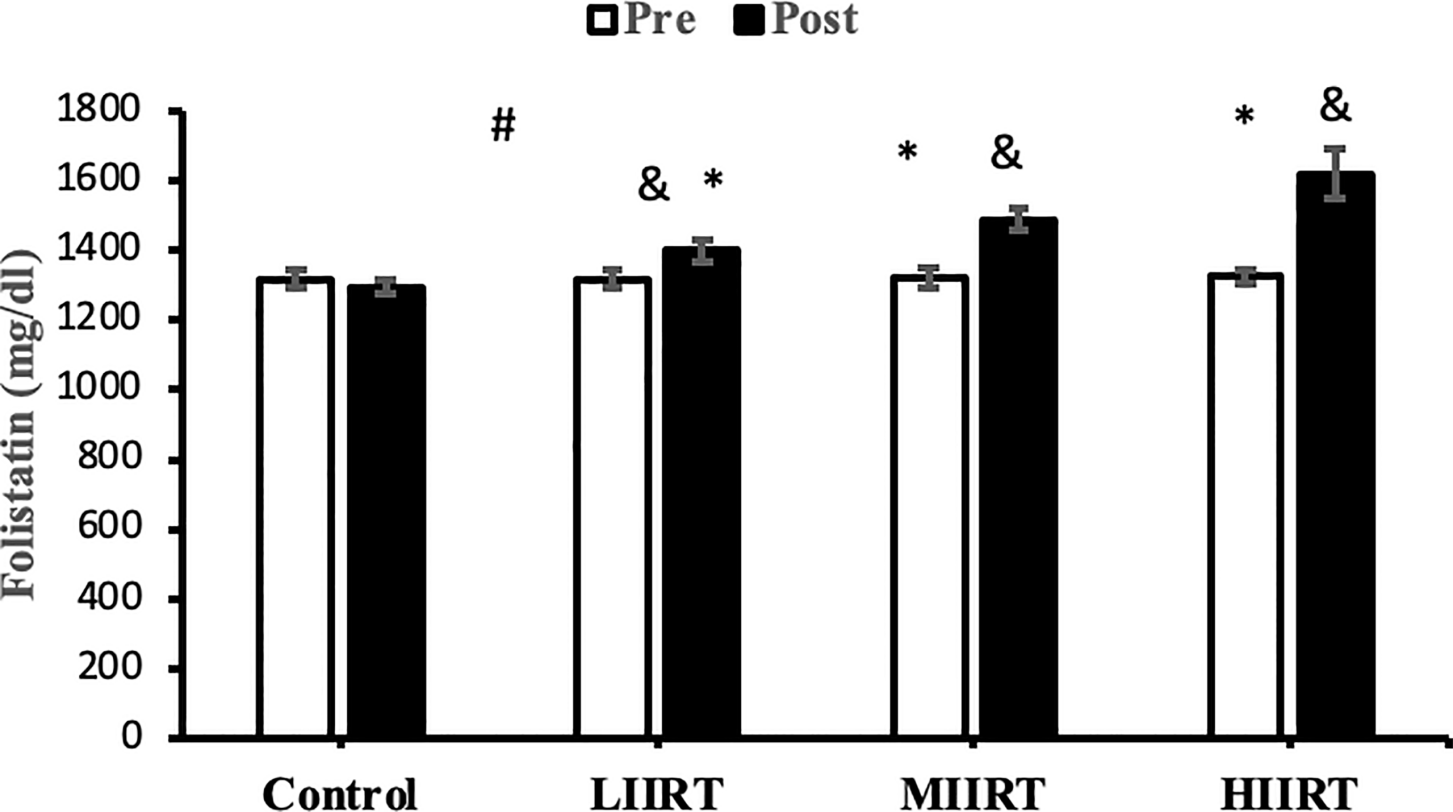
Pre and post-training values (mean ± SD) for follistatin in control, low-intensity interval resistance training (LIIRT), moderate-intensity interval resistance training (MIIRT), high-intensity interval resistance training (HIIRT) groups. *Significant differences from the control group (p<0.05). ^&^Significant differences from pretest values (p<0.05). ^#^Significant differences between training groups (p<0.05).

**Figure 5:**
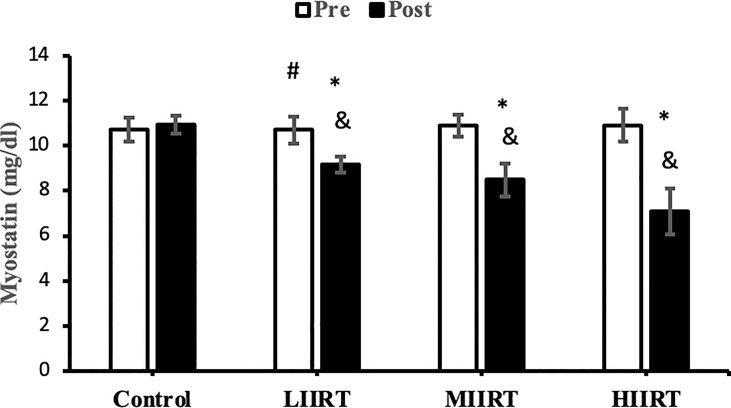
Pre and post-training values (mean ± SD) for myostatin in the control, low-intensity interval resistance training (LIIRT), moderate-intensity interval resistance training (MIIRT), high-intensity interval resistance training (HIIRT) groups. *Significant differences from the control group (p<0.05). ^&^Significant differences from pretest values (p<0.05). ^#^Significant differences between training groups (p<0.05).

**Figure 6:**
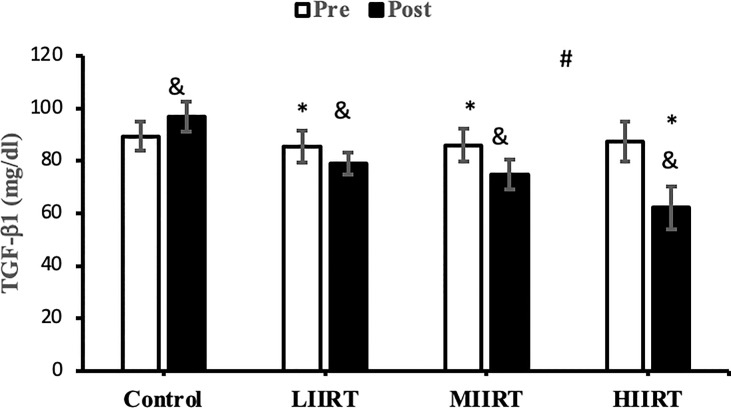
Pre and post-training values (mean ± SD) for TGF-β1 in control, low-intensity interval resistance training (LIIRT), moderate-intensity interval resistance training (MIIRT), high-intensity interval resistance training (HIIRT) groups. *Significant differences from the control group (p<0.05). ^&^Significant differences from pretest values (p<0.05). ^#^Significant differences between training groups (p<0.05).

There were no differences in baseline levels of decorin (p=0.89), activin A (p=0.29), follistatin (p=0.90), myostatin, (p=0.73) and TGF-β1 (p=0.47) between the groups. There were significant group X time interactions for decorin (p=0.0001, η^2^ = 0.81), activin A (p=0.0001, η^2^ = 0.49), follistatin (p=0.0001, η^2^ = 0.85), myostatin (p=0.0001, η^2^ = 0.71), and TGF-β1 (p=0.0001, η^2^ = 0.65) (**Figures 2**–**6**).

Post-test values for decorin, follistatin, and myostatin differed from pre-test values in LIIRT, MIIRT, and HIIRT groups (p<0.05) but not in the control group (p>0.05). Pre-post differences in activin A were not significant in the control (p=0.10) and LIIRT (p=0.26) groups, while pre-post differences for TGF-β1 were significant in all four groups (p<0.01). *Post-hoc* analysis of differences between groups showed that the changes of plasma decorin in the control group were significantly different from changes in MIIRT (p=0.001) and HIIRT (p=0.001) but not in LIIRT (p=0.12). The increase in plasma decorin levels was significantly greater in HIIRT than MIIRT (p=0.01) and in MIIRT than LIIRT (p=0.001). *Post-hoc* analysis showed that changes of activin A in the control group were different from changes in plasma activin A in MIIRT (p=0.001) and HIIRT (p=0.001) but not in LIIRT (p=0.12). Changes of activin A in HIIRT were significantly different from changes in LIIRT (p=0.007) and in MIIRT in comparison to LIIRT (p=0.001), but not in HIIRT in comparison to changes of activin A in MIIRT (p=0.95). *Post-hoc* analysis showed that the changes of follistatin in LIIRT, MIIRT, and HIIRT were significantly different from changes of follistatin in the control group (p<0.05), while changes of follistatin in HIIRT were greater than LIIRT (p=0.008) and in MIIRT than LIIRT (p=0.0001), but not in HIIRT compared to MIIRT (p=0.32). *Post-hoc* analysis revealed that changes in myostatin levels in the LIIRT, MIIRT and HIIRT groups were different from changes in the control group (p<0.05), and there were also differences between LIIRT and HIIRT (p=0.0001) and also MIIRT and HIIRT (p=0.008) but not between LIIRT and MIIRT (p=0.18). *Post-hoc* analysis revealed that changes of TGF-β1 in LIIRT, MIIRT, and HIIRT were different from changes in the control group (p<0.05). The reduction in TFG-β1 was greater in the HIIRT group than in the MIIRT (p=0.003) and LIIRT (p=0.0001) groups, but with no significant differences between the LIIRT and MIIRT groups (p=0.62).

## Discussion

This study investigated the effects of three different interval resistance training (IRT) protocols and their effects on anthropometric indices, cardiometabolic markers, and levels of decorin, follistatin, myostatin, activin A, TGF-β1 in men with obesity. Repeated exercise sessions improve cardiometabolic health by inducing changes in myokines or proteins/hormones (53). We demonstrated that 12 weeks of LIIRT, MIIRT, and HIIRT improved lipid profiles (LDL, TC, TG), glucose, insulin, HOMA-IR, weight, BMI, fat mass, FFM, VO_2peak_, decorin, follistatin, TGF-β1, and myostatin, with no changes in VO_2peak_, HDL, or activin A levels in the LIIRT group.

The effects of resistance training on TGF-β1 have not been investigated in humans. Chronic exercise improves the thermogenic capacity to protect against body fat accumulation and other metabolic diseases by suppressing the TGF‐β1/IkB‐α axis in the hypothalamus and causing an over expression of uncoupling protein (UCP1) in obese mice (54). Eight weeks of exercise reduces TGF-β levels in skeletal muscle in middle-aged participants while decreasing mitochondrial oxidation and insulin sensitivity (55). TGF-β1 has important roles in metabolism and cellular responses to mechanical loading of various tissues (56). Levels of TGF-β1 correlate with VO_2peak_, BMI, fat mass, adiposity, insulin resistance, and other metabolic disorders in humans (12) and is a negative regulator of metabolic adaptation to exercise training by inhibiting insulin signaling, mitochondrial activation, and enzymes involved in optimal metabolic adaptations in response to training (57). Circulating levels of TGF-β1 are increased in *ob/ob* mice and obese humans (58). Therefore, the decreases in TGF-β1 in the present study are likely related to increases in decorin (59), follistatin (59), FFM (12), VO_2peak_ and HDL (12), and the decrease in LDL, BMI, fat mass (12), myostatin and activin A (59).

Follistatin regulates muscle mass, glucose metabolism, and insulin resistance, and plasma levels of follistatin are increased by band resistance training (60), with greater increases produced by HIIT than with RT (61). The mechanisms of follistatin secretion are unknown, but mechanical load, muscle contraction, and stretching of contractile proteins lead to the release of myogenic factors such as follistatin (62). Increases in circulating follistatin levels due to muscle contraction result from increased expression of hepatic follistatin mRNA and protein following exercise (63). Increasing the ratio of glucagon to insulin in hepatocytes leads to the release of follistatin into the bloodstream. The decrease in serum insulin levels in our study suggests that follistatin release increased from the liver as an adaptation to interval resistance training. On the other hand, changes in IL-6 are reported to have an indirect effect on follistatin secretion; IL-6 is a communicating signal between skeletal muscle cells and the liver, whose secretion increases up to 100-fold during muscle contraction and lack of energy (64). We can speculate that the type of interval resistance training protocol in our study (which could deplete energy levels in the muscles) likely caused IL-6 to indirectly up-regulate follistatin levels. Others have reported that exercise-induced follistatin levels have similar effects as insulin-like growth factors secreted by the liver in response to growth hormone (63). Many functions of follistatin are facilitated through natural inhibition of other transforming growth factors such as activin and myostatin (65). Therefore, the reasons for the increase in follistatin in the present study are increases in decorin (59), FFM (12), VO_2peak,_ and HDL (12) and decreases in TGF-β1 (59, 65), LDL, BMI, fat mass (12), myostatin and activin A (59).

Decorin is secreted from myotubes in response to exercise and plays a role in the exercise‐related restructuring of skeletal muscles in healthy young men (66). Decorin inhibits myostatin-induced inhibition of muscle growth to enhance the proliferation and differentiation of myoblasts (67) and stimulates myogenic satellite cell proliferation and differentiation by regulating cellular responsiveness to TGF-β1, resulting in muscle hypertrophy (68). Therefore, the increase in decorin in the present study may be due to increases in follistatin (59), FFM (12), VO_2peak,_ and HDL (12) and decreases in TGF-β1 (59, 65), LDL, BMI, fat mass (12), myostatin and activin A (59). The present study, in addition to being a novel study that examines the effect of different intensities of IRT on myokine changes and metabolic risk factors, also The results of our study confirmed the findings of other studies on the importance of myokine changes on the physiological and metabolic status of the body (12, 57, 59, 65).

Resistance exercise increases muscle mass, strength, and power to improve secondary metabolic profiles (69). HIRT is an atypical RT protocol where participants use heavy loads to induce mechanical effects on muscle with very short recovery periods comparable to high-intensity endurance training such as HIIT. The intensity of exercise is one of the regulators of muscle growth factors. The rate of release of myokines and regulators of muscle growth is dependent on the intensity and volume of muscles involved in exercise (62). The intensity and level of resistance affect the activation rate of motor units. Therefore, the amount and type of motor units involved in the activity affect the levels of secretory myokines and their autocrine and paracrine function (70). The recruitment of more fast-twitch motor units, which occurs as a result of high-intensity training compared to low-intensity training, has a greater effect on the levels of muscle myokines as well as the signaling rate of myogenic factors (70, 71). Our results suggest that a short, intense training protocol that improves myokines and cardiovascular risk factors may be an attractive alternative to more traditional and time-consuming exercise training methods (72).

## Limitations

Our study is not without limitations: (1) The lifestyles and dietary intakes of participants were not controlled, although they were instructed by a nutritionist and were asked to maintain the same diet throughout the study, (2) We used bioelectrical impedance to measure body composition variables and acknowledge that bioelectrical impedance is not the gold standard method for measuring such compositional markers; however, its reliability and validity have been reported in numerous other studies, (3) The RT protocols used in our study were not equalized according to calorie expenditure, which might have influenced the results, (4) Our study only included male participants, and finally (5) The group sample sizes were relatively small and as such, added to the variability in our data.

## Conclusion

We found that MIIRT and HIIRT caused greater increases in circulating levels of decorin, follistatin, and HDL than LIIRT. In addition, both MIIRT and HIIRT also had positive effects on insulin resistance, insulin, and LDL levels. Our findings suggest that MIIRT and HIIRT may be more useful approaches for reducing metabolic indices associated with cardiovascular disease in sedentary individuals with obesity. However, additional studies are needed to substantial our findings as well as determine the time courses of changes in these myokines produced by exercise since our study was limited to a comparison of pre- and post-training.

## Data Availability Statement

The original contributions presented in the study are included in the article/supplementary material. Further inquiries can be directed to the corresponding authors.

## Ethics Statement

The studies involving human participants were reviewed and approved by Research and Ethics Committee of the Islamic Azad University (Ethics code: IR-IAU1397-16). The patients/participants provided their written informed consent to participate in this study.

## Author Contributions

AA, AS, and HZ conceived and designed research. AA, AS, HA, HR, AD, ZP, VH, GB, and AM conducted experiments and analyzed data. AA, AS, and HZ wrote the manuscript. IL, MA, AH, TV, and HZ revised the manuscript. All authors read and approved the manuscript.

## Funding

Funding for this publication is supported by Kennesaw State University.

## Conflict of Interest

The authors declare that the research was conducted in the absence of any commercial or financial relationships that could be construed as a potential conflict of interest.

## Publisher’s Note

All claims expressed in this article are solely those of the authors and do not necessarily represent those of their affiliated organizations, or those of the publisher, the editors and the reviewers. Any product that may be evaluated in this article, or claim that may be made by its manufacturer, is not guaranteed or endorsed by the publisher.
